# The Practicality of the Use of Liquid Biopsy in Early Diagnosis and Treatment Monitoring of Oral Cancer in Resource-Limited Settings

**DOI:** 10.3390/cancers14051139

**Published:** 2022-02-23

**Authors:** Henry Ademola Adeola, Ibrahim O. Bello, Raphael Taiwo Aruleba, Ngiambudulu M. Francisco, Tayo Alex Adekiya, Anthonio Oladele Adefuye, Paul Chukwudi Ikwegbue, Fungai Musaigwa

**Affiliations:** 1 Department of Oral and Maxillofacial Pathology, Faculty of Dentistry, University of the Western Cape, Tygerberg Hospital, Cape Town 7505, South Africa; 2 Division of Dermatology, Department of Medicine, Faculty of Health Sciences and Groote Schuur Hospital, University of Cape Town, Observatory, Cape Town 7925, South Africa; 3 Department of Oral Medicine and Diagnostic Sciences, College of Dentistry, King Saud University, Riyadh 11545, Saudi Arabia; ibello@ksu.edu.sa; 4 Department of Molecular and Cell Biology, Faculty of Science, University of Cape Town, Cape Town 7700, South Africa; arlrap001@myuct.ac.za; 5 Grupo de Investigação Microbiana e Imunológica, Instituto Nacional de Investigação em Saúde (National Institute for Health Research), Luanda 3635, Angola; franciscongiamb@yahoo.com; 6 Wits Advanced Drug Delivery Platform Research Unit, Department of Pharmacy and Pharmacology, School of Therapeutic Science, Faculty of Health Sciences, University of the Witwatersrand, Parktown, 7 York Road, Johannesburg 2193, South Africa; adekiyatalex@gmail.com; 7 Division of Health Sciences Education, Office of the Dean, Faculty of Health Sciences, University of the Free State, P.O. Box 339, Bloemfontein 9300, South Africa; adefuyeao@ufs.ac.za; 8 Division of Immunology, Faculty of Health Sciences, Institute of Infectious Diseases and Molecular Medicine (IDM), University of Cape Town, Cape Town 7925, South Africa; ikwpau001@myuct.ac.za (P.C.I.); msgfun001@myuct.ac.za (F.M.)

**Keywords:** Africa, cfDNA, circulating tumour cells, exosomes, liquid biopsy, oral squamous cell carcinoma

## Abstract

**Simple Summary:**

Mouth cancer often results in poor outcomes and requires the use of state-of-the-art medical approaches to make its detection easy, individualized, and early. Liquid biopsy is a new and important medical approach to disease detection. This approach has been successfully used for mouth cancer detection and monitoring of treatment progress in many countries. Liquid biopsy is an attractive option for mouth cancer detection because it does not involve any invasive procedure and can be used on easily accessible body fluids, such as saliva and blood. Furthermore, there is evidence that this technology has some advantage over the normal tissue biopsy because it is not invasive, neither does it need any surgical expertise. Hence, we have focused on how easy or practical it would be, to employ the use of liquid biopsy on the African continent, as well as other low- and middle-income countries. We have discussed the different types of this technology in three main areas of focus, *viz*, what factors are important before, during and after collection of samples for liquid biopsy analysis, and what are the obstacles to routine use of this approach in resource-limited settings.

**Abstract:**

An important driving force for precision and individualized medicine is the provision of tailor-made care for patients on an individual basis, in accordance with best evidence practice. Liquid biopsy(LB) has emerged as a critical tool for the early diagnosis of cancer and for treatment monitoring, but its clinical utility for oral squamous cell carcinoma (OSCC) requires more research and validation. Hence, in this review, we have discussed the current applications of LB and the practicality of its routine use in Africa; the potential advantages of LB over the conventional “gold-standard” of tissue biopsy; and finally, practical considerations were discussed in three parts: pre-analytic, analytic processing, and the statistical quality and postprocessing phases. Although it is imperative to establish clinically validated and standardized working guidelines for various aspects of LB sample collection, processing, and analysis for optimal and reliable use, manpower and technological infrastructures may also be an important factor to consider for the routine clinical application of LB for OSCC. LB is poised as a non-invasive precision tool for personalized oral cancer medicine, particularly for OSCC in Africa, when fully embraced. The promising application of different LB approaches using various downstream analyses such as released circulating tumor cells (CTCs), cell free DNA (cfDNA), microRNA (miRNA), messenger RNA (mRNA), and salivary exosomes were discussed. A better understanding of the diagnostic and therapeutic biomarkers of OSCC, using LB applications, would significantly reduce the cost, provide an opportunity for prompt detection and early treatment, and a method to adequately monitor the effectiveness of the therapy for OSCC, which typically presents with ominous prognosis.

## 1. Introduction

Reaching the goal of early and accurate diagnosis, as well as providing the best evidence-based treatment option for disease, is a key driving force for precision and individualized medicine [1]. A plethora of diagnostic methods have already been developed and, recently, liquid biopsy (LB) has shown increased global interest as a precision diagnostic tool in the field of cancer research. Cancer as a major global public health problem is emerging as a critical concern in Africa, where many cancer cases are diagnosed at late stages of the disease. This is due to factors such as limited knowledge and expertise of disease screening, limited diagnostic infrastructure, and for most patients, fear of surgery, poverty, lack of access to specialist care, and low educational level, among other factors—these are some of the key barriers to early presentation and cancer diagnosis among African populations [2]. 

Novel diagnostic tools such as LB could help in addressing these challenges due to its non-invasive nature, accuracy, and the fact that it does not require surgical facilities. LB has emerged as a rapid, reliable, and minimally invasive cancer screening solution, with high specificity and sensitivity for cancer diagnosis and monitoring. As demonstrated in developed countries, the high specificity and sensitivity of LB offers a promising diagnostic tool that would enhance screening capability and the potential for the early diagnosis of cancer cases in Africa; as well as probably reduce the incidence of morbidities and mortalities from cancer on the continent. In addition, the implementation of policies that allow easy access to valuable cancer diagnostic procedures, such as LB, can further help reduce the financial burden of late cancer management in many low-income countries in Africa. 

Oral squamous cell carcinoma (OSCC) ranks amongst the ten most prevalent cancers in the world with high morbidity and mortality rates [3]. This emphasizes the need for, and the importance of, screening programs and techniques for the early detection of malignancy. A lack of access to oral health care, which can lead to a delay in diagnosis has been reported to decrease the survival rates of OSCC in several low and middle-income countries including those in Africa [4]. The timely detection and diagnosis of OSCC may save lives by improving the survival rate, reducing treatment-related morbidities and improving the surveillance of recurrent cancer cases in these countries [5]. Therefore, it is vital to further understand how LB procedures are currently emerging and are used in low-and middle-income countries and, most importantly, to understand the blood biomarkers involved in oral cancer.

## 2. Liquid Biopsy and Cancer Management

For centuries, the use of tissue biopsies in cancer has enabled the histological characterization of the disease. Its application has provided insights into the genetic profile of tumors, allowing for good cancer management [6]. Notably, tissue biopsy remains the gold standard for diagnostic analyses in clinical settings. However, tissue biopsies involve invasive surgical techniques, cost, and tissue sample preparation. More importantly, tissue biopsies may not capture genetic heterogeneity within a tumor, and in intermetastatic tumor samples, thus affecting the accuracy of the test [7]. These challenges described make LB an appealing alternative, especially as a useful instrument in long-term management and prognostication. 

LBs can be used to investigate biological components in liquid forms in cancer patients for diagnosis, screening, and prognosis. LB may involve the analysis of released circulating tumor cells (CTCs) and circulating tumour DNA (ctDNA) in the blood or body fluid of a cancer patient [8]. These analytes are complementary biomarkers that present great potential for various cancer drug discovery platforms. Other analytes that can also be identified by using LB include circulating cell-free RNA (cfRNA), exosomes and platelets [9]. Liquid biopsy analytes can improve our understanding of tumor heterogeneity, and provide potentially better cancer diagnosis, treatment, and surveillance, as well as detecting drug resistance. Importantly, saliva, urine, pleural effusions, seminal plasma, sputum, cerebrospinal fluid, and stool samples are other physiological fluids that can be utilized for a LB in addition to blood [10,11].

### 2.1. Circulating Cell-Free DNA (cfDNA) and Circulating Tumor DNA (ctDNA) in OSCC

Fragmented, tumor-derived DNA that are not associated with cells (i.e., cell-free) and which are found within the circulatory system are known as ctDNA. They are the tumor derived part of circulating cell-free DNA (cfDNA), which refers to the total DNA shed into the blood and biological fluids during apoptosis and necrosis under physiologic and pathologic conditions [12]. Currently, ctDNA has been used in monitoring the therapeutic response and the detection of cancer relapse at early stages. Regarding treatment response and relapse detection, the identification of tumor-specific point mutations, promoter hypermethylations, and the identification of allelic imbalance using microsatellite markers analysis in ctDNA are helpful tools of assessment in patient management [13]. The ctDNA possesses short nucleic acid fragments of around 166 bp located in the plasma [14]. Healthy individuals have lower levels of cfDNA when compared with OSCC patients, which further increase as the cancer metastasizes, indicating its simultaneous use as a promising diagnostic and prognostic biomarker [15]. However, increased cfDNA is not specific for cancer, and in individual patients, there is no specific cut-off value which can be attributed to the tumor in quantification as ctDNA. This limitation can be overcome by evaluating tumor specific alterations such as methylations and mutations [16]. The potential for using ctDNA in all stages of head and neck squamous cell carcinoma (HNSCC) diagnosis and management was highlighted in a recent review—this included screening and early detection; prognosis and the detection of minimal residual disease; the characterization of mutational landscape; precision medicine and treatment selection; and treatment monitoring and identification of resistant clones, based on experience with HNSCC of other sites using ctDNA analysis [17]. Several studies have been developing techniques to detect cfDNA (or ctDNA), but there is still a lack of a standardized method, which is essential for its clinical application together with the need to reduce the cost of analysis. Studies have identified ctDNA as a diagnostic biomarker and this has been used in many diseases [18]. Although these studies demonstrated that circulating cfDNA can screen for cancer; when testing for ctDNA, the procedure requires additional investigation and an acceptable cost before these can be applicable for use as a diagnostic or therapeutic tool, especially in African and other resource-limited settings.

### 2.2. Circulating Tumor Cells (CTCs) in OSCC

CTCs are released into circulation by the primary tumor (i.e., they are of tumor origin) as a result of spontaneous or iatrogenic factors. They are believed to share a similar profile with the somatic mutations and genomic rearrangements present in the primary tumor [19]. They are able to reflect the tumor heterogeneity, which may be missed in surgical biopsies. This makes them good candidates for understanding tumor mutational profiles without allowing patients to go through invasive tissue biopsy. A major problem with CTC’s use in patient management is their extremely low number in peripheral circulation (1–100 CTCs per 1 billion peripheral blood cells). They need to be enriched and separated for detection and analysis by often tedious and expensive methods [20,21]. Increasing levels of CTCs have been reported to be associated with poor prognosis and distant metastasis in several forms of cancer. Survival rate in OSCC, and other cancers such as breast, lung, prostate and ovarian cancer, has been linked with levels of CTCs, indicating their benefit in the early screening and treatment monitoring of cancer [22]. Partridge et al. [23] evaluated the levels of disseminated tumor cells preoperatively and intraoperatively in both blood and bone marrow from 40 patients with OSCC. They found a high risk of loco-regional recurrence and distant metastasis associated with the presence of CTCs. In 2015, Oliveira-Costa et al., provided more knowledge regarding CTC biology for OSCC by analyzing the gene expression profile of OSCC tumors to identify biomarkers that decreased or increased during tumor progression [24]. Their results showed that programmed death-ligand 1 (PD-L1), HOXB9 and ZNF813 expression in OSCC-derived CTCs was increased, while B Cell Linker (BLNK) expression decreased. In summary, the investigators reported that PD-L1 is a prognostic factor in OSCC as expressed in patients CTCs and provides insights for the development of an anti-PD-L1 therapy for OSCC patients. Due to PD-L1 inducing an exhaustion state in T-cells and reducing the capability of a T-cell-mediated response, they hypothesized that OSCC patients could benefit from anti-PD-L1 therapy. Therefore, the results reported in this study emphasized the role of CTCs as an independent prognostic marker in OSCC. Recurrent assessments of CTC levels have also been used in studies to demonstrate the potential utility of CTCs in disease monitoring before, during, and after therapy, Inhestern et al. [25] examined and evaluated CTC counts in blood samples from 40 patients with OSCC. Apart from its potential as a prognostic biomarker, there is interest in investigating the role of CTCs in regulating disease behavior. The checkpoint inhibitors that block the PD-1/PD-L1 immune checkpoint pathway on CTCs and stimulate the immune system to remove CTCs in circulation may reduce the risk of metastasis and disease recurrence. In patients with OSCC, PD-L1 overexpression in CTCs was identified and utilized to monitor the treatment response [24]. Numerous studies [26,27,28,29,30] showed that LB procedures, such as CTCs, can be utilized as a cutting-edge technology that may improve the detection and monitoring of cancer using a small amount of blood samples. CTCs assessment using LB as a cancer biomarker promises to be low cost for the management of patients with OSCC. 

### 2.3. Exosomes in OSCC

Exosomes are bioactive vesicles with diameters ranging between 40–150 nm that are used for analyzing LBs. A miRNA expression profile has shown that circulating exosomal miR-21 was associated with hypoxic tumor and metastasis in the lymph node in people with OSCC [31]. The detection of miRNA biomarkers in both the plasma and tumors of patients with squamous cell carcinoma of the tongue highlights the significance of free and exosomal miRNAs as potential diagnostic biomarkers for tongue cancer. In addition, packaged circulating miRNAs in protein complexes or encapsulated within microvesicles are protected against the activity of blood RNAses, and represent a more dependable approach for the assessment of circulating tumor-miRNA signatures [10]. More so, exosomes in the tumor microenvironment have been implicated in increasing levels of the transforming growth factor-B (TGF-β) pathway; thus, increasing drug resistance and tumor growth in OSCC patients. Exosomal chemokine-like factor (CKLF)-like MARVEL transmembrane domain-containing 6 (CMTM6) of OSCC cells aid the polarization of alternatively activated macrophages (M2) via activation of the signaling of ERK1/2 in macrophages [32]. Indeed, the classically activated macrophages possess anti-tumor properties; conversely, the M2 functions as a pro-tumor, aiding in the development and progression of the tumor [33]. Exosomes are released by different types of cells into numerous biological fluids such as amniotic fluid, cerebrospinal fluid, lymph, bile, ascites, tears, breast milk, urine, semen, blood, and saliva, both in healthy and diseased conditions [34,35,36,37]. Two research groups have demonstrated that exosomes are present in the tumor microenvironment, demonstrating its importance in tumorigenesis, tumor invasion, and metastasis, since they can possess an anti-tumor function or promote tumor progression [38,39]. In OSCC, exosomes have been shown to be key components in the tumour microenvironment, increasing the TGF signaling pathway, which contributes to the progression and drug resistance of OSCC [40]. Limited available evidence may suggest a potential discriminatory biomarker role of exosomes, between active OSCC disease patients and cured OSCC patients. In addition, a study by Zlotogorski-Hurvitz et al. [41] morphologically characterized oral fluid-derived exosomes in OSCC. The potential scope of the diagnostic and prognostic application of exosomes in oral cancer has been described elsewhere [32]. Additionally, the role of some exosomal miRNA (e.g., miR-223, miR-101-3p, miR-338 and miR-34a-5p) as tumor suppressors and the robust potential of exosomes for therapeutic drug delivery to the tumor for effective treatment or to improve prognosis has been highlighted in another recent review [34].

### 2.4. Messenger Ribonucleic Acid in OSCC

LBs find and evaluate several biomarkers, such as mRNA biomarkers, pro-inflammatory cytokines, and metabolites in the saliva, urine, and plasma of OSCC patients. mRNA biomarkers from saliva for use in the early diagnosis of oral cancers were recently described by Oh and colleagues [35]. Thirty candidate genes related to cancer previously reported in the literature were selected. Thirty-three OSCC patients and 34 non-tumor controls had their saliva samples taken and the mRNA levels of six genes CYP27A1, NAB2, collagen type III alpha 1 (COL3A1), monoamine oxidase B (MAOB), nuclear pore complex interacting protein B4 (NPIPB4), and sialic acid acetyltransferase (SIAE) were considerably lower in the saliva of OSCC patients. The combination of SIAE and CYP27A1 had an AUC of 0.84, which was considered good. In the under 60 group, the AUC of MAOB–NAB2 was more prognostic of OSCC (AUC, 0.91; specificity, 0.86; and sensitivity, 0.92) than any other transcript combination. The results from this study suggested salivary mRNAs were useful biomarkers for early OSCC diagnosis, especially in individuals under 60 years old. Lu et al. [36] found substantially increased expression of plasma miR-196a and miR-196b in patients with cancerous and precancerous oral cavity lesions, with excellent sensitivity and specificity compared to the normal controls. Furthermore, Liu et al. [37] found a substantial increase in plasma miR-31 in OSCC patients, and a significant decrease following tumor excision, indicating that miRNAs might be used for diagnostic and treatment monitoring purposes.

### 2.5. Saliva as a Liquid Biopsy Substrate

Saliva allows for a non-invasive LB; it is easily accessible and has a large number of biomarkers for illnesses, as well as pre-symptomatic and health status indicators. The saliva biofluid contains RNA and DNA molecules, cytokines, extracellular vesicles (EVs), and circulating and tissue-derived cells, which are novel biomarkers or indicators [38,39,40]. Salivary biomarkers may be effective for the early detection of OSCC since they are physically accessible to the mouth cavity [38]. In saliva and tissue samples from individuals with OSCC and oral potentially malignant diseases, NF-κB-dependent cytokines, and pro-inflammatory cytokines (IL-6, IL-1α, IL-8, and TNF-α) were assessed [41]. NF-κB-dependent cytokines, matrix metalloproteinases (MMPs) and pro-inflammatory cytokines in saliva may be involved in the relationship between oral malignancies and aging, involving the senescence-associated secretory phenotype (SASP), and inflammatory diseases, such as oral mucosal ulcers and periodontitis [42]. Metabolome biomarkers in OSCC were also investigated using nuclear magnetic resonance (NMR) metabolomics to detect high-risk patients with an extranodal extension [43]. Recent research has revealed that EVs, including exosomes (small vesicles) and bigger vesicles, are released into the saliva from oral cancer lesions and/or oral tissues [44]. Meanwhile, many biomarker proteins of oral malignancies, such as heat shock proteins (HSPs) [45,46,47], and epidermal growth factor receptor (EGFR) [48], have been discovered in EVs. Thus, microRNAs in EVs of saliva could also be important molecules for early detection of oral malignancies distinct from aging [49], periodontitis [49,50], and state of health [51].

### 2.6. Novel Molecular Techniques for Application of Liquid Biopsy

More sensitive emerging technologies are now employed for LB analysis. These technologies include beads, emulsion, amplification, and magnetics (BEAMing), digital droplet PCR (ddPCR) and next generation sequencing (NGS) [36]. In respect of experimental and clinical applications, BEAMing, ddPCR and NGS have separate applications, and they sometimes complement each other for the examination of a tumor at molecular level [36]. ddPCR is very reliable and accurate for the investigation of genetic alteration in various cancers due to its very sensitive nature [37]. However, this method is challenged by a low multiplexing capacity, but efforts are in the pipeline towards designing multiplexed strategies to reduce experimental artefacts [37]. LBs can be used to monitor OSCC treatment responses and tumor evolution in real-time and improve the prognostic and diagnostic potential for OSCC [10,38]. One of the essential benefits of analyzing LBs in OSCC is that they give a tailored snapshot of primary and metastatic tumors at different periods, allowing clinicians to detect early signs of disease recurrence or resistance to therapy and assisting them in their therapeutic decisions [10]. As a result, by employing LBs, a molecular profile for each patient can be acquired. Different tumor subtypes may be useful in supplementing the tumor, node, and metastasis (TNM) staging approach [10]. A summary of the applications of LB in OSCC can be found in Table 1.

## 3. The Challenges of Utilizing Liquid Biopsy as an OSCC Diagnostic and Management Tool in Africa: Pre-Analytic, Analytic Processing, and Post-Analytic Processing Phase Considerations

Despite the progress made by oral LB in different parts of the world, its practical application in African and other resource-limited settings remains a major challenge. Basically, tumor components, including ctDNA, ctRNA, CTCs and/or extracellular vesicles (exosomes), shed into the circulation from the tumor site, are increasingly becoming clinically useful in the detection and monitoring of tumor or cancer progression [10]. These biopsies are employed for early detection of the circulating tumor origins from bodily fluid. However, the process of employing these biopsies has been faced with several challenges, which have hampered their optimal treatment applications. For instance, although oral swabbing can be used for tumor surveillance after treatment, it is very difficult to apply this screen to a high-risk population without tumor relevant lesions.

Moreover, oral sample may not be useful in differentiating the origin of the tumor cells in the circulation [52]. In addition, cfDNA comprises mutated and non-mutated tumors and normal DNA; thus, depending on the types and stages of the tumor, differentiating ctDNA from the mix could be burdensome. In some instances, a very small amount (≤0.01%) of ctDNA is used for variant genotyping. This often occurs where there is an extremely low frequency of CTCs, making it difficult to detect ctDNA at the early stage of cancer, and further complicating the detection and recovery processes [30]. 

The inability to differentiate and specifically target tumor components or associated biomarkers from already existing non-tumor cells in circulation is a critical issue facing all LBs, which might have delayed their optimal clinical use [53]. For example, trying to use ctDNA to capture the full heterogeneity of cancer cells may also introduce the possibility of capturing DNA of normal cells [54], which may consequently increase the chances of error and false-positive results. Due to the heterogeneity of tumor cells, there is a likelihood of genetic alteration of the CTCs detected in bio-fluid circulation compared to the original tumor profile, decreasing the accuracy of the finding [55]. LB may never reach its full potential because detecting CTCs (which is the major biomarker targeted during a LB) requires a huge amount of blood or complex enrichment media to achieve an optimal result and an acceptable level of specificity and sensitivity [56]. 

Regarding the LBs, specificity and sensitivity cannot be overemphasized as they are critical to validating the result’s accuracy, efficacy, and reliability. In the assessment of CTCs in LB, the use of epithelial cells as sole biomarkers for the enrichment of this assay poses a serious challenge, especially when epithelial cells have lost their physical characteristics and specificity after undergoing epithelial-to-mesenchymal-transition (EMT) and gained mesenchymal properties [57]. This makes it almost impossible for them to be detected by the typical methods of CTC isolation; hence, compounding the issues of sensitivity and specificity of LB. Again, the reported high level of epithelial cells in the circulation of patients suffering from benign diseases is an indication that the use of the epithelial cell adhesion molecule (EpCam) to capture CTC may introduce unwanted errors and misdiagnosis [58]. Sample collection, storage, shipping, and transportation are critical for the stability of the collected specimen. If they are not well handled, it could lead to the degradation of the sample, hence producing varying results. The Ethylenediaminetetraacetic acid (EDTA) used to maintain the longevity and stability of blood LBs has a high incidence of genomic DNA contamination when stored for a long time [59]. Due to poor transport facilities in the major parts of African countries, it is unlikely that the reagent would reach the rural communities in good condition, which would hamper its clinical application efficacy. The reproducibility issue of biomarkers is due to the lack of standardization guidelines that give rise to the variability of results as reported in vesicle counting of extracellular vesicles, which reduces the reliability and reproducibility. Therefore, the results may not be reproducible or comparable in various study cohorts [60].

In an African context, many obstacles could prove to be wedging blocks that prevent the practical implementation of LB in OSCC in this continent. Thus, so far, only three African countries have adapted and authorized or commercialized the use of these techniques in the public domain. These countries are South Africa, Tunisia, and Kenya, and this could be due to weighing the financial burden of cost, approximately US$7000 per test, which can only be afforded by high-income earners who form less than 10% of the population of most African countries [61]. It will become more challenging to implement in low- and middle-income countries in sub-Saharan Africa with a Gross Domestic Product (GDP) of less than US$15 billion. Moreover, considering that most of the available LBs have not been tested in African populations, it may be harder for physicians to ascertain their efficacy in Africa due to environmental and genetic variations. In addition to the inadequate infrastructural facilities, technical know-how, limited skilled personnel, poor government policy, a lack of many adequate research institutes, and religious and cultural practices have all slowed scientific innovation and progress in Africa [62]. Further, the coronavirus pandemic has negatively impacted the economy of many African countries and this could further delay the practical implementation of oral LB in Africa. 

Furthermore, despite its evidence-based theranostic potential to improve cancer management, LB is prone to several analytical discrepancies, which until resolved, could limit its applicability in clinical practice. Inconsistencies and errors may be notable at any of the stages in the workflow, leading to sample misevaluation. These methodological inconsistencies may pose a risk to patients due to a discrepancy in cancer management and the unnecessarily high costs to patients, laboratories, and hospitals [63]. Therefore, consistency in a clinical setting is critical for optimal patient safety, the accuracy and precision of laboratory tests and, overall, the routine applicability of LB in clinical settings.

### 3.1. Pre-Analytic Phase Considerations

In the pre-analytical phase, a substantial part of the sample workflow occurs outside a controlled laboratory setting [64,65]. Previous reports have highlighted that this phase is subject to the most errors in the sample evaluation workflow [63], and could limit the integrity of the sample and the data quality [66,67]. Study-specific variations in ctDNA assays and insufficient ctDNA recovery limit the validation of plasma ctDNA based LBs for cancer screening under a clinical setup [68]. Multiple studies have demonstrated that the quality of cfDNA and ctDNA could be impacted by the type of sample collection tube used, optimal storage conditions, and the time-lapse between sample collection and sample processing [65,69,70,71]. As a result, the optimization of sample collection and processing is dependent on the type of collection tube used and is critical for optimal downstream data output. Serum isolating standard EDTA tubes have been linked with leukocyte lysis that could increase genomic DNA concentration and potentially reduce the ease of access to ctDNA, which is often available in small quantities [64,65,72]. To minimize the risk of genomic DNA contamination and dilution of ctDNA and cfDNA, optimizing the time-lapse between sample collection and the initiation of sample processing, as well as the sample storage temperatures during transportation is essential [71]. Genomic DNA contamination of cfDNA could be minimized by sampling whole blood [65], and potentially saliva, in cfDNA stabilizing collection tubes containing fixatives. These tubes could improve the recovery of high-quality cfDNA with the added advantage of a prolonged storage time and variable shipping temperature [71,72,73]. However, these specialized tubes could be more costly than the EDTA tubes and, therefore, may not be easily implementable in resource-constrained settings such as in developing African nations. 

### 3.2. Analytic Processing Phase Considerations

Similar to the pre-analytical stage, sample processing is a consolidated effort of many laboratory aspects aiming to produce high-quality clinical data. Easily identifiable is a varying range of methodologies including reagents and applied technologies (including different instruments) used by separate laboratories to achieve the same goal of generating high-quality clinical data [74]. Different laboratories may use different ‘clinically validated’ consumables yet with differing clinical results, particularly when different commercial suppliers are used for supposedly similar clinical assessments [75]. This poses the risk of differing and possibly incorrect interpretations of clinical data, and thus puts patients at risk of incorrect cancer management plans [75].

Several LB analytical methods with varying sensitivities and specificities are prone to laboratory-specific variations [65]. In the processing phase, detection technologies used to process LB samples are prone to variations that could raise inconsistencies. In addition, most of these technologies are expensive and cannot be afforded by most low- and middle-income countries in Africa.

#### 3.2.1. Next Generation Sequencing (NGS)

Taking NGS assays as examples, despite their notable success in advancing the application of LBs in clinical settings, technical heterogeneity of these assays within the same studies or amongst different studies have been described as potential limitations to the implementation of NGS in routine clinical practice [76]. In addition, there are no consensus-based standard molecular coverage parameters affecting the sensitivity for NGS. Under these conditions, data output interpretation is often site-specific, with a heightened risk of false-negative results [76,77,78]. 

#### 3.2.2. Digital Droplet PCR (ddPCR)

Similarly, ddPCR has been praised for its analytical strengths over real-time quantitative PCR [79] in addition to its high sensitivity, which is comparable to deep sequencing [80,81]. However, except for intralaboratory validations, no consensus-based analytical and clinical validation guidelines for cfDNA assays on ddPCR exist [79]. Consequently, the implementation of this technology is often site or laboratory-specific, and its output could be potentially variable across several laboratories. Inconsistencies in ddPCR could, therefore, influence the interpretation of the output data, resulting in inconsistent patient management depending on the site of the clinical sample evaluation. As such, these inconsistencies require careful control through expert agreement, standardized validation, and optimization practices in clinical settings [65,77,82].

The use of these technologies need to be refined and tailored to address the operational challenges, which currently limit their application in Africa [83]. The financial burden of using these molecular technologies deserves some consideration. The cost concerning technical and staff equipment, including a variety of professionals such as molecular and computational biologists, genetic counselors, and specialized clinicians, are considerably high and can be unaffordable for many low-income countries on the continent [84].

### 3.3. Post-Processing Quality Check, Data Analysis and Bioinformatics Considerations

**Figure 1:**
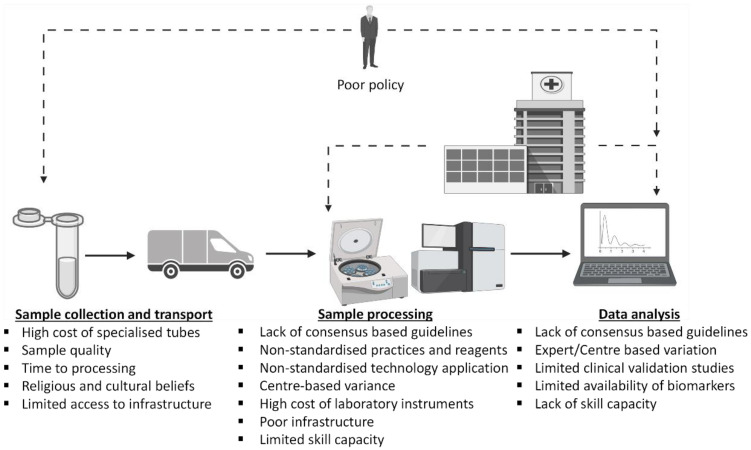
Challenges associated with the implementation of liquid biopsy in poorly resourced settings. Created in BioRender.com, accessed on 21 July 2021).

The optimization of molecular data analyses would improve reproducibility while maintaining high-quality clinical inferences. Laboratory protocol and analytical workflow standardization remain the cornerstone of clinical laboratory practice to ensure consistently reproducible and accurate clinical data output for optimal patient management in OSCC cancer care [85]. The reproducibility and reliability of laboratory results can be improved by the standardization of sample processing workflow, through expert consensus. These can be achieved via large-scale clinical trials, which validates the critical steps required to improve the reliability, accuracy, and consistency of clinical data at acceptable methodology specificity and sensitivity levels [82,86]. Moreover, supporting the need for further research validation, LB in oral cancer is an emerging field that is still limited by the lack of knowledge on sensitive and specific circulating biomarkers for oral cancer detection [10]. Altogether, it is imperative to establish clinically validated working guidelines for various aspects of LB sample collection, processing, and analysis for optimal and reliable use of LBs and their associated technologies in routine clinical care (see Figure 1).

## 4. Future Perspectives of Liquid Biopsy in Africa

LB has emerged as a potential non-invasive approach in precision, personalized cancer medicine, particularly for OSCC. Different studies have demonstrated the benefit of OSCC LB, using various downstream analysis such as CTCs, cfDNA, miRNA, mRNA, Long non-coding RNA (lncRNA) and salivary exosomes, and have shown promise for early OSCC diagnosis, treatment monitoring, and detection of occult disease and relapse [2]. However, many of these downstream LB analyses are yet to be routinely applied in clinics for oral cancer. In Africa, and especially in sub-Saharan countries, the impact of LBs in the clinical setting is still very limited, requiring further research for effective implementation. Further discovery of a robust panel of sensitive and specific circulating biomarkers for OSCC at different stages would help, not only policymakers in decision-making, but also clinicians to improve diagnosis and prognosis. A better understanding of OSCC biomarkers would be key for the development of effective therapies for the management of oral cancer. The continent needs to quickly improve the management of oral cancer, and since LB for OSCC is in its infancy, researchers working in oral cancer should put their efforts into performing large, but also prospective multicenter studies that investigate the role of CTCs, ctDNA, cfDNA, miRNA, incRNAs and exosomes in oral cancer. Better use of LBs in oral cancer can help in the monitoring and surveillance for post-treatment recurrence as well as the detection of new cancer cases. Thus, due to the cross-cutting low socio-economic status of populations, Africa needs a reproducible and non-invasive LB that could provide the basis for individualized diagnosis, therapeutic strategies, and precision management of OSCC. Although the advantages of LBs in the evaluation of OSCC prognosis, risk stratification, or its use as a tool for precision medicine development, has been discussed here, a very challenging aspect is its reproducibility across multiple centers, to establish a standard guideline for LB-based cancer diagnostics. The development of LB databases such as liqDB [87], ctcRbase [88,89], and BloodPAC Data Commons (BPDC) [90], has helped to overcome such technical issues and has encouraged data science research to address the practical and conceptual reproducibility problems associated with the use of LB [91]. Hence, the development of an OSCC LB database in Africa and other resource-limited settings, could potentially ameliorate the diagnostic and treatment burden of OSCC in these regions.

## Figures and Tables

**Table 1:** Some evidence of the applications of liquid biopsy in oral cancer.

Study	Country	Type of Cancer	Liquid Biopsy Technology	Publication Type	Reference
Lousada-Fernandez et al., 2018	Spain	Oral cancer	Liquid Biopsy	Review	[10]
Lin et al., 2018	Taiwan	Oral Cancer	Cell-Free DNA Biomarker	Original Research	[15]
Patel et al., 2016	India	Oral Squamous Cell Carcinoma	Circulating Tumor cells	Original Research	[19]
Economopoulou et al., 2017	Greece	Head and Neck Squamous Cell Carcinoma	Circulating Tumor Cells	Review	[20]
Oliveira-Costa et al., 2015	Brazil	Oral Squamous Cell Carcinoma	Circulating Tumor Cells	Original Research	[24]
Inhestern et al., 2015	Germany	Oral and Oropharyngeal Squamous Cell Cancer	Circulating Tumor Cells	Original Research	[25]
Li et al., 2016	China	Oral Squamous Cell Carcinoma	Exosomes	Original Research	[31]
Pang et al., 2021	China	Oral Squamous Cell Carcinoma	Exosomes	Original Research	[32]
Lu et al., 2021	China	Oral Squamous Cell Carcinoma	Exosomes	Review	[34]
Oh et al., 2020	South Korea	Oral Cancer	Salivary mRNA Biomarker	Original Research	[35]
Lu et al., 2015	Taiwan	Oral Cancer	Circulating miRNA Biomarker	Original Research	[36]
Liu et al., 2010	Taiwan	Oral Cancer	Circulating miRNA Biomarker	Original Research	[37]
Cristaldi et al., 2019	Italy	Oral Squamous Cell Carcinoma	Salivary Biomarker (ctDNA, EVs and miRNAs)	Review	[38]
Adeola et al., 2020	South Africa	Oral Cancer	Salivary Exosomes Biomarker	Review	[40]
Tsai et al., 2020	Taiwan	Oral Squamous Cell Carcinoma	Nuclear Magnetic Resonance Metabolomics Biomarker	Original Research	[43]
Ono et al., 2018	Japan	Oral Cancer	HSP-Enriched Properties of Extracellular Vesicles	Original Research	[45]
Fujiwara et al., 2018	Japan	Oral Cancer	Exosomes	Original Research	[48]
Spafford, et al., 2001	USA	Head and Neck Squamous Cell Carcinoma	Pretreatment Oral Rinse Microsatellite Analysis.	Original Research	[52]

## Data Availability

The data presented in this study are available in article.
